# Including Dominance Effects in the Genomic BLUP Method for Genomic Evaluation

**DOI:** 10.1371/journal.pone.0085792

**Published:** 2014-01-08

**Authors:** Motohide Nishio, Masahiro Satoh

**Affiliations:** NARO Institute of Livestock and Grassland Science, Tsukuba, Japan; University of California, Riverside, United States of America

## Abstract

We evaluated the performance of GBLUP including dominance genetic effect (GBLUP-D) by estimating variances and predicting genetic merits in a computer simulation and 2 actual traits (T4 and T5) in pigs. In simulation data, GBLUP-D explained more than 50% of dominance genetic variance. Moreover, GBLUP-D yielded estimated total genetic effects over 1.2% more accurate than those yielded by GBLUP. In particular, when the dominance genetic variance was large, the accuracy could be substantially improved by increasing the number of markers. The dominance genetic variances in T4 and T5 accounted for 9.6% and 6.3% of the phenotypic variances, respectively. Estimates of such small dominance genetic variances contributed little to the improvement of the accuracies of estimated total genetic effects. In both simulation and pig data, there were nearly no differences in the estimates of additive genetic effects or their variance between GBLUP-D and GBLUP. Therefore, we conclude GBLUP-D is a feasible approach to improve genetic performance in crossbred populations with large dominance genetic variation and identify mating systems with good combining ability.

## Introduction

Genomic selection refers to the use of genome-wide dense single nucleotide polymorphism (SNP) markers to predict breeding values and subsequently select individuals [1]. Several approaches of genomic prediction have been presented. One of them is the genomic best linear unbiased prediction (GBLUP), which uses genomic information in the form of a genomic relationship matrix that defines the additive genetic covariance between individuals [2], [3]. The genomic relationship coefficients are estimated with higher accuracy than when using pedigree information because genomic information can capture of Mendelian sampling across the genome. GBLUP has become popular approach in genomic selection of dairy cattle [4], [5] because it is simple and has low computational requirements [6], [7].

Most published models only include additive genetic effects [8], and little research has been performed to expand these models to predict genetic merits to account for dominance genetic effects. It can be argued that such expansion is difficult because calculation becomes complicated and de-regressed estimated breeding values are used as phenotypes in most applications of genomic selection [9]. However, dominance genetic effect is of theoretical and practical important because it is heavily used in crosses of animal breeds. In fact, assortative mating and mate allocation boost the field performances of livestock [10]. Genomic selection has, therefore, renewed the interest in the prediction of dominance genetic effects. For example, the dominance genetic variance accounted for 5.6% of the phenotypic variance by GBLUP including dominance genetic effect [11]. More recently, GBLUP method including dominance genetic effect was suggested and the software (GVCBLUP) are already available online (http://animalgene.umn.edu/) [12]–[14].

The present study aimed to evaluate the performance of GBLUP including dominance genetic effect by estimating variance components and predicting genetic effects for both simulation and actual pig data.

## Materials and Methods

### Stochastic Simulation

A historical population was simulated to establish mutation drift equilibrium. The simulated genome comprised one chromosome 1 Morgan long that contained 6,000 SNP markers and 300 randomly spaced biallelic quantitative trait loci (QTL). In the first generation of the historical population, the initial allele frequencies of all markers and QTL were assumed to be 0.5. The recurrent mutation process was applied, and the mutation rate of markers and QTL was 5.0×10^−4^ per locus per generation. Recombinations were sampled from a Poisson distribution with a mean of 1 per Morgan and were then randomly placed along the chromosome. The historical population evolved over 2,000 generations of random mating and random selection with a population size of 100 (50 males and 50 females) to reach mutation–drift balance.

After 2,000 historical generations, a base population (G0) and the subsequent 6 generations (G1 to G6) were generated as a recent population. The population size of G0 increased to 300 (150 males and 150 females). In G1 to G6, 30 sires were randomly selected and mated to 150 dams in each generation. Each dam had 1 son and 1 daughter; thus, each sire had 5 sons and 5 daughters.

In G0, 1,000 markers and 50 QTL were randomly selected among the segregating markers and QTL with minor allele frequencies >0.05. Let 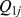 and 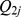 be 2 alleles at the *j*th QTL. The genetic values are then given by 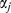, 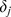, and 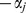 for genotypes 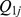 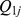, 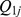 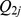, and 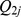 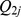, respectively. The value of 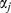 was drawn from a gamma distribution with a shape parameter of 0.42; its sign was drawn at random with equal chance. According to the previous simulation study including dominance genetic effects [15], the value of 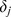 was determined as the product of the absolute of 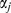 and the degree of dominance, which was drawn from a normal distribution 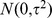. The total genetic effect (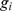) of the *j*th animal was calculated by summing all QTL genotypic values, and its variance (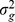) was calculated as the sum of additive and dominance genetic variances (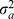 and 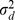) [16], which were calculated as follows:
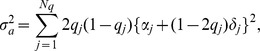


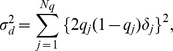
where 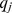 is the frequency of 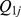. The broad-sense heritability (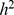) of the trait was 0.3. To obtain phenotypic values, an environmental effect was added to the total genetic effect, which was sampled from a normal distribution 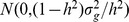.

The phenotypes and genotypes of SNP markers were available for 1,500 and 1,800 individuals from G1 to G5 and G1 to G6, respectively. Thus, the reference population with both phenotypes and genotypes comprised 1,500 individuals from G1 to G5, and the test population with only genotypes comprised 300 individuals in G6.

In a standard simulation scenario, 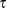 and the number of markers in G0 (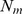) were set to 0.5 and 1,000, respectively. To investigate the effects of 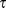 and 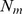 on the performance of the present method, 2 alternative scenarios were simulated in addition to the standard scenario. Three different values of 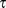 (0.25, 0.5, and 1.0) and 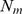 (200, 1,000, and 5,000) were simulated in the first and seconds groups, respectively. For all of these alternatives, only the intended parameter differed from the standard scenario. Twenty replicates were simulated for each scenario.

### PIC Pig Data

Publicly available data including pedigree, genotypic, and phenotypic information on a single Pig Improvement Company (PIC) nucleus pig line were used (http://www.g3journal.org/content/2/4/429/Suppl/DC1). The total number of individuals was 3,512, and all have phenotypes for 2 traits (T4 and T5) and genotypes available from the PorcineSNP60 chip (*N = *64,233). These phenotypes were already adjusted for environmental fixed effects such as sex, farm, and year of birth [17]. Then, 1,800 individuals were randomly selected from all individuals whose accuracies from the full PIC dataset exceeded 0.8. From these 1,800 individuals, 1,500 were selected from old generations and defined as the reference population while the other 300 individuals were defined as the test population. Genotypes were filtered for minor allele frequencies less than 0.05. The pig data are summarized in Table 1.

**Table 1:** Summary of PIC pig dataset.

		Number of animals		Phenotype		Accuracy of estimated breeding value from all PIC datasets	
Trait	No. SNPs	Reference	Test	Mean	SD	Mean	SD
T4	27,391	1,500	300	−1.125	2.417	0.875	0.048
T5	27,287	1,500	300	44.107	60.315	0.880	0.048

### Genomic BLUP Model

The GBLUP including additive and dominance genetic effects termed GBLUP-D. The statistical model of GBLUP-D can be expressed as:
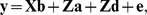
where 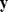 is the vector of phenotypes; 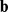 is the vector of fixed effects; 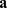 and 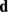 are the vector of additive and dominance genetic effects of animals; 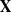 and 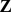 are incident matrices for the fixed effects, additive, and dominance genetic effect, respectively; and 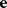 is the vector of residuals. Additive and dominance genetic effects were assumed to follow normal distributions: 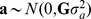 and 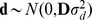, where 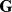 and 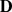 are additive and dominance genomic relationship matrices, respectively. These matrices describe the relationships between genotyped individuals and can be constructed from the information on genome-wide SNP markers. Let 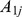 and 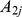 be 2 alleles at the *j*th marker locus and 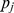 be the frequency of 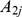. The 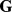 matrix is created as follows [3]:



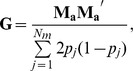
where 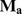 is the 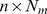 matrix (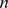 is a number of individuals) and the element of 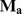 for the *i*th individual at the *j*th marker is calculated as follows:



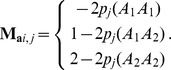



Similarly, 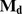 is assumed to be the 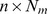 matrix and the element of 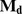 for the *i*th individual at the *j*th marker can be calculated as:
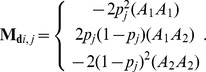



This element describes the coefficients of 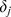 in dominance deviations. Therefore, 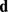 and its variance can be derived as follows:
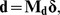
where 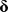 is the 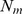 dimensional vector of the *j*th element, which is 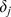. This dominance formula was also used in GVCBLUP [14]. Assuming the dominance genetic effects at different marker loci are identically and independently distributed normal variables, the variance of the genome-wide dominance effect is calculated as follows:



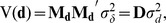


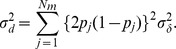



Consequently, 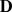 can be calculated using 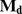:
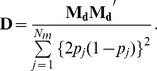



Variance components were estimated with average information restricted maximum likelihood (REML) [18]. The dataset of reference population were used to predict genetic effects of the genotyped individuals in test population. The GBLUP-D model solutions yielded the estimates of additive (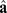) and dominance genetic effects (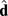). The estimates of the total genetic effect (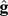) were calculated by the sum of 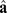 and 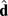. In GBLUP, 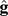 equals 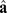, because the dominance genetic effect is not considered. The predictive ability of the model was evaluated from accuracy and unbiasedness of estimates in the test population. The accuracies of estimated additive, dominance, and total genetic effects (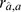, 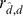, and 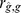, respectively) were measured as the correlations between the estimates and true values. Unbiasedness (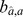, 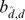, and 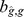) was measured using the regressions of estimates on true values. A regression coefficient of one denotes unbiasedness. Since the true values are unknown in pig data, estimated breeding values from the full PIC dataset (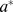) and phenotypes (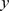) were used instead of true additive and total genetic effects, respectively. In real data, the predictive ability of dominance genetic effect cannot be calculated and was inferred from that of total genetic effect.

## Results

### Stochastic Simulation

Tables 2 and 3 show the estimates of variance components and heritability in simulation data with various values of 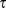 and 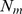. For all values of 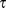 and 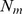, there were nearly no differences in estimates of additive genetic variance and narrow-sense heritability between GBLUP and GBLUP-D. The ratios of dominant genetic variance estimated by GBLUP-D were 90.6%, 61.1%, and 54.4% with a 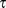 of 0.25, 0.5, and 1.0 and 46.6%, 61.1%, and 84.0% with an 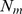 of 200, 1,000, and 5,000, respectively.

**Table 2:** Variance component estimates (±standard errors) and heritabilities for simulation data with 3 dominance degrees (0.25, 0.5, and 1.0).

		Genetic variance components				
Condition	Method	Additive	Dominance	Residualvariance	Narrow-senseheritability	Broad-senseheritability
*τ* = 0.25	True value	0.259	0.032	0.736	0.267	0.299
*N_m_* = 1,000	GBLUP	0.277±0.036	–	0.793±0.022	0.256	–
	GBLUP-D	0.277±0.036	0.029±0.012	0.764±0.024	0.259	0.286
*τ* = 0.5	True value	0.191	0.108	0.736	0.185	0.289
*N_m_* = 1,000	GBLUP	0.205±0.029	–	0.865±0.024	0.192	–
	GBLUP-D	0.208±0.030	0.066±0.016	0.797±0.026	0.194	0.256
*τ* = 1.0	True value	0.138	0.147	0.706	0.139	0.290
*N_m_* = 1,000	GBLUP	0.152±0.024	–	0.857±0.024	0.151	–
	GBLUP-D	0.152±0.024	0.080±0.016	0.773±0.025	0.151	0.231

**Table 3:** Variance component estimates (±standard errors) and heritabilities for simulation data with 200, 1,000, and 5,000 markers.

		Genetic variance components				
Condition	Method	Additive	Dominance	Residualvariance	Narrow-senseheritability	Broad-sense heritability
*τ* = 0.5	True value	0.195	0.103	0.739	0.188	0.287
*N_m_* = 200	GBLUP	0.158±0.026	**–**	0.878±0.024	0.153	**–**
	GBLUP-D	0.158±0.026	0.048±0.012	0.831±0.024	0.152	0.199
*τ* = 0.5	True value	0.191	0.108	0.736	0.185	0.289
*N_m_* = 1,000	GBLUP	0.205±0.029	**–**	0.865±0.024	0.192	**–**
	GBLUP-D	0.208±0.030	0.066±0.016	0.797±0.026	0.194	0.256
*τ* = 0.5	True value	0.211	0.075	0.685	0.217	0.295
*N_m_* = 5,000	GBLUP	0.206±0.027	**–**	0.761±0.021	0.213	**–**
	GBLUP-D	0.207±0.028	0.063±0.016	0.698±0.024	0.214	0.279

Tables 4 and 5 show the accuracies and unbiasedness of estimated genetic values calculated by GBLUP and GBLUP-D in simulation data. For all values of 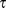 and 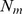, 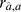 and 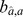 were almost equal between GBLUP-D and GBLUP. In GBLUP-D, 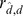 and 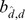 increased with increasing 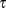 and 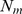. Meanwhile, 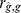 values in GBLUP-D exceeded those in GBLUP by 1.2%, 7.8%, and 24.7% with a 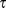 of 0.25, 0.5, and 1.0 and by 1.9%, 7.8%, and 4.2% with an 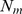 of 200, 1,000, and 5,000, respectively. In GBLUP-D, larger values of 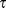 resulted in 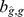 being closer to 1.

**Table 4:** Accuracies of estimates (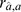, 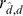, and 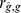) and regression coefficients of estimates on their true values (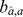, 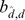, and 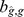) in the test population for simulation data with 3 dominance degrees (0.25, 0.5, and 1.0).

Condition	Method	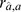	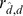	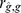	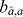	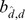	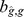
*τ* = 0.25	GBLUP	0.803	–	0.760	0.898	–	0.939
*N_m_* = 1,000	GBLUP-D	0.804	0.212	0.769	0.902	0.609	0.942
*τ* = 0.5	GBLUP	0.743	–	0.616	0.891	–	0.976
*N_m_* = 1,000	GBLUP-D	0.745	0.339	0.664	0.900	0.893	0.994
*τ* = 1.0	GBLUP	0.711	–	0.466	1.001	–	0.937
*N_m_* = 1,000	GBLUP-D	0.712	0.478	0.581	1.006	1.189	1.035

**Table 5:** Accuracies of estimates (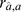, 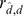, and 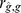) and regression coefficients of estimates on their true values (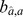, 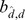, and 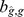) in the test population for simulation data with 200, 1,000, and 5,000 markers.

Condition	Method	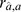	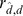	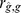	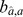	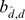	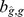
*τ* = 0.5	GBLUP	0.689	–	0.552	1.049	–	1.027
*N_m_* = 200	GBLUP-D	0.696	0.246	0.563	1.062	0.547	0.938
*τ* = 0.5	GBLUP	0.743	–	0.616	0.891	–	0.976
*N_m_* = 1,000	GBLUP-D	0.745	0.339	0.664	0.900	0.893	0.994
*τ* = 0.5	GBLUP	0.799	–	0.694	1.004	–	1.035
*N_m_* = 5,000	GBLUP-D	0.801	0.374	0.723	1.010	0.961	1.032

### PIC Pig Data

In T4 and T5 from the pig data, dominance genetic variance accounted for 9.6% and 6.3% of the phenotypic variance, respectively (Table 6). The estimated additive genetic variances and residual variances calculated by GBLUP-D were smaller than those calculated by GBLUP. Thus, GBLUP-D consequently yielded lower narrow-sense heritability and higher broad-sense heritability than GBLUP.

**Table 6:** Variance components estimates (±standard errors) and heritabilities for PIC pig data.

		Genetic variance components				
Trait	Method	Additive	Dominance	Residual variance	Narrow-sense heritability	Broad-sense heritability
T4	GBLUP	1.909±0.189	**–**	3.678±0.138	0.342	**–**
	GBLUP-D	1.735±0.201	0.537±0.219	3.331±0.185	0.310	0.405
T5	GBLUP	1298.1±123.2	**–**	2198.2±84.3	0.371	**–**
	GBLUP-D	1239.0±129.3	220.8±125.9	2049.3±113.1	0.353	0.416

In T4 and T5 from the pig data, there were nearly no differences between GBLUP and GBLUP-D with respect to 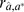
(Table 7). In T5, 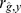 and 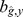 were slightly higher in GBLUP-D than GBLUP, whereas 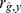 and 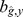 were not higher in GBLUP-D in T4.

**Table 7:** Aces of estimates (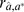
and 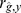) and regression coefficients (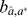 and 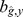) of 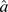 on full PIC dataset (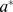) and 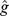 on phenotypic value (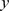) in the test population for the PIC pig dataset.

Trait	Method	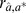	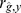	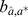	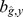
T4	GBLUP	0.455	0.286	0.637	0.726
	GBLUP-D	0.456	0.286	0.710	0.724
T5	GBLUP	0.379	0.288	0.631	0.786
	GBLUP-D	0.376	0.299	0.654	0.805

## Discussion

### Stochastic Simulation

The GBLUP-D method captured the substantial ratios of the dominance genetic variances and estimated the individual dominance genetic effects although there were nearly no differences in estimates of additive genetic variance and narrow-sense heritability between GBLUP and GBLUP-D. Our result indicates that GBLUP-D is expected to improve performance of the crossbreds, in particular when degree of dominance is large.

In the present study, the simulated genome comprised one chromosome 1 Morgan long. However, the whole genome sizes of livestock are larger. Here, another simulation data were constructed to evaluate the effect of the genome size on predictive ability. This simulation data comprised five chromosomes of 1 Morgan. The numbers of markers and QTL set to be 5,000 and 250 to obtain the same distances between markers and QTL as the initial simulation. Table 8 shows the accuracies and unbiasedness of estimated genetic values in this simulation data. The dominance genetic effects could be captured in this data, but the accuracies of additive, dominance and total genetic effects decreased in comparison with the genome of 1 chromosome. Hence, large size of reference population would be required when genome size is large.

**Table 8:** Accuracies of estimates (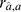, 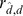, and 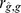) and regression coefficients of estimates on their true values (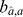, 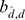, and 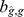) in the test population for simulation data with 3 dominance degrees (0.25, 0.5, and 1.0) when genome comprises of 5 chromosomes with 1 Morgan each.

Condition	Method	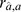	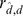	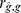	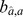	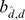	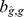
*τ* = 0.25	GBLUP	0.672	–	0.636	1.012	–	1.017
*N_m_* = 1,000	GBLUP-D	0.673	0.148	0.641	1.013	0.810	1.018
*τ* = 0.5	GBLUP	0.646	–	0.502	1.015	–	1.018
*N_m_* = 1,000	GBLUP-D	0.647	0.244	0.528	1.016	1.011	1.015
*τ* = 1.0	GBLUP	0.612	–	0.490	1.112	–	1.124
*N_m_* = 1,000	GBLUP-D	0.612	0.348	0.525	1.122	1.030	1.120

### PIC Pig Data

The degrees of estimated dominance genetic variance in T4 and T5 were nearly equal to those in simulation data with a 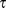 of 0.25. When 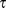 was 0.25, 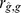 and 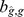 were 1.2% and 0.3% higher in GBLUP-D than GBLUP. These results indicate the predictive ability of GBLUP-D in T4 and T5 only improved slightly because the degrees of dominance genetic variance were too small. In fact, 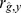 and 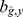 in GBLUP-D were little improved in comparison with GBLUP. In general, the degree of dominance genetic variance is expected to be much larger in crossbred populations than purebred ones. Since the present study was based on data from purebred PIC pig data, the degrees of dominance genetic variance in T4 and T5 might have been small.

### Practical Use of GBLUP-D

GBLUP-D has two practical uses. First, selection on the basis of GBLUP-D in crossbreds is useful for commercial production. In swine and poultry, crossbreds are the end product. The marker information from a purebred and its crossbred relatives enables the selection of candidate purebreds for the performance of their crossbred offspring [11]. Second, GBLUP-D could allow mating allocation to exploit dominance. An extra response will be obtained when an appropriate design of future matings using mating allocation techniques is implemented [10], [19].

### Additive and Dominance Relationship Matrix

In the presence of dominance genetic effects, the breeding values of 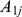 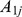, 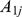 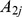, and 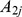 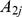 at the *j*th locus are 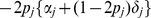, 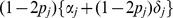, and 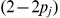 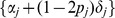, respectively. The additive genomic relationship matrix in GBLUP-D should be constructed considering 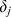. If all QTL are of complete dominance, then 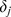 is 1. However, in practice, the value of 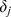 cannot be determined because the degree of dominance is unknown. Therefore, in the present study, the additive genomic relationship matrix was the same in GBLUP-D and GBLUP. Although there are nearly no differences in the estimates of additive genetic effects or their variance between GBLUP and GBLUP-D, the additive genomic relationship matrix including 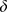 may yield good estimates of them.

A GBLUP method including dominance genetic effects was also proposed in a previous study [11]. In that model, the additive genomic relationship matrix is same as that in traditional GBLUP. However, the dominance genomic relationship matrix (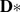) in the previous study differs from that in the present study and is defined as follows:
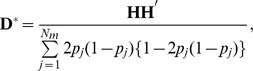
where the element of 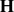 for the *i*th individual at the *j*th marker is calculated as



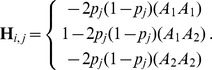



This element corresponds to the heterozygosity coefficient but not the dominance genetic effect. To compare the performance of GBLUP-D in the present study and the model in the previous study [11], predictive ability was calculated in the standard simulation scenario (Table 9). GBLUP-D yielded higher accuracies of additive, dominance, and total genetic effects than the previous model [11]. This might be because the heterozygosity coefficient includes part of the additive genetic effect. In fact, the previous study [12] reported that dominance genetic variance calculated from the previous model [11] was larger than that from GBLUP-D.

**Table 9:** Accuracies of estimates (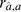, 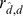, and 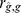) and regression coefficients of estimates on their true values (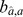, 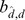, and 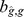) in the test population for simulation data (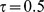,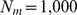).

Method	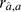	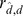	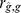	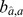	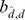	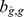
GBLUP	0.743	–	0.616	0.891	–	0.976
GBLUP-D	0.745	0.339	0.664	0.900	0.893	0.994
Su et al. [17]	0.709	0.239	0.655	0.939	0.569	0.967

Assuming linkage equilibrium and uncorrelated marker effects, the dominance genetic variances in the present study (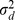) and the previous study [11] (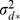) are calculated as follows:
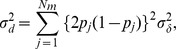


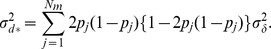



In addition, the estimated dominance genetic effects in the present study (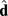) and the previous study [11] (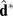) are calculated as follows:
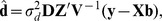


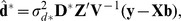
where 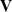 is the variance of 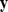. If the distribution of the allelic frequencies is available, 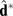 can be transformed to 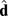.

### Epistasis

Increasing knowledge about biological pathways and gene networks highlights the importance of gene–gene interactions, i.e., epistasis; some authors argue that much of the genetic variance in a population is due to such interactions [20]–[22]. When considering second-order epistasis in GBLUP, the epistatic genomic relationship matrix can be approximately calculated from the Hadamard product of the genomic relationship matrix. For example, additive by additive and additive by dominance interactions are represented as 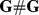 and 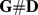, respectively. The present study tried to use linear mixed models including 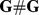 and 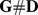 for T4 and T5 in pig data. However, the variance components of these epistasis could not be estimated because they were outside parameter space (data not shown). The previous study [11] also included epistatic genomic relationship matrices in GBLUP. In that model, the estimated epistatic variances were almost 0 for daily gain in Danish Duroc pigs. The estimated epistatic variances in the Bayesian model for the percentage of CD8^+^ cells in publicly available mouse data were almost 0 [23]. Although these studies could not detect epistastic effect, in recent, the marker-generated kinship matrices were suggested in a new mixed model method [24] and nonparametric approaches and machine-learning techniques were recommended to model more complex gene interaction patterns [25]–[27].
